# Supplementary material for: Implicit racial biases are lower in more populous more diverse and less segregated US cities
Source: Nat Commun. 2024 Feb 6;15:961. doi: 10.1038/s41467-024-45013-8 (PMC10847142; doi:10.1038/s41467-024-45013-8)
Supplement: Supplementary file 1 — Supplementary Information [file 41467_2024_45013_MOESM1_ESM.pdf]

Supplementary Note

Here we reproduce the parts of our previously developed extension to the standard Urban Scaling Theory model [1] that are relevant for inter-group interactions and implicit racial biases.

The extension on which this manuscript is based starts from the standard formulation of Urban scaling theory [2, 3], which describes cities as spatially embedded networks of socioeconomic interactions.

For the average per-capita number of social interactions, k, the urban scaling relationship takes form of $k \sim N^{\delta}$, where $\delta = \frac{1}{6}$. This form of the scaling relationship results from a mean-field approximation that individuals interact homogeneously. Under these conditions, we take individuals to have an interaction cross section a_0 and a characteristic travel length l per unit time. This gives the average number of interactions for a large city ($N >> 1$) as :

$$k \sim \frac{a_0 l}{A_n} N \tag{1}$$

, where A_n is the area of the city's networks. The scaling relationship for the area of the city's networks, $A_n \sim N^{1-\delta}$ [2], recovers the scaling relationship for k.

In order to derive the model for heterogeneous group interactions it is important to understand the standard formulation of the scaling relationship for interactions. We start by observing that, $\frac{a_0 l}{A_n}$ is a probability composed of the fraction of a city's area that individuals cover, on average, over a given time period. This is the average probability of interacting with other individuals in the city. Thus, the total expected number of interactions for each individual is given by their probability of interacting, $\frac{a_0 l}{A_n}$, multiplied by the number of individuals they could interact with, N.

Next, we extend this standard formulation by modeling each individual in the city as belonging to some distinct number of groups, indexed by g. Individuals in these groups may interact with a lower probability with other groups. We define this relative reduction in out-group inter-

actions by $1 - h_g^{bet}$, where $h_g^{bet} \in [0,1]$ is the segregation of group g.

With these definitions, the average number of interactions for individuals in group g with individuals in different groups is given by:

$$k_{g,inter} \sim \frac{a_0 l}{A} \sum_{j \neq g} N_j (1 - h_g^{bet}) \tag{2}$$

where N_g is the population of focal group g. The total number of between-group social interactions for all individuals in group g is $k_{g,inter} N_g$, on average. Therefore, the average number of between-group social interactions for individuals in an observed segregated city i with G different groups, is $k_i \sim \frac{1}{N} \sum_{g=1}^{G} k_{g,inter,i} N_{g,i}$.

This brings us almost to Equation 2 of the main text:

$$k_{i,inter} \sim \frac{a_0 l}{A_i N_i} \sum_{j \neq g} N_{j,i} (1 - h_{g,i}^{bet})) \cdot N_{g,i} \tag{3}$$

Multiplying and dividing through by N_i^2 we have:

$$k_{i,inter} \sim \frac{a_0 l N_i^2}{A_i N_i} \sum_{j \neq g} \frac{N_{j,i}}{N_i} \frac{N_{g,i}}{N_i} (1 - h_{g,i}^{bet})) \cdot \tag{4}$$

which directly simplifies to the between-group interactions terms of Equation 2 of the main text:

$$k_{i,inter} \sim N^{\delta} \sum_{j \neq g} \frac{N_{j,i}}{N_i} \frac{N_{g,i}}{N_i} (1 - h_{g,i}^{bet})) \cdot \tag{5}$$

since $\frac{a_0 l N_i}{A_i} \sim N^{\delta}$.

Note that when segregation is complete, i.e., $h_{g,i}^{bet} = 0$ or there is only one group in the city, $k_{i,inter}$ goes to 0, as expected, and there are no between-group interactions.

As a note, in general, segregation values need not be the same across all groups and we can define a matrix with entries $\in [-1,1]$ which specifies the degree to which individuals avoid or preferentially interact with individuals from the same or other groups:

$$H_i = \begin{bmatrix} h_{11i} & h_{12i} & \cdots & h_{1Gi} \\ h_{21i} & h_{22i} & \cdots & h_{2Gi} \\ \vdots & \vdots & \ddots & \vdots \\ h_{G1i} & h_{G2i} & \cdots & h_{GGi} \end{bmatrix} \tag{6}$$

The relative rate of interactions between any two groups (or within a group) is given by $1 + H_i$. In the model presented here, we constrain H so that each group avoids all other groups equally and that there are repeated off-diagonal entries that are all negative. Specifically, that $h^{bet}_{g,i} = h_{gji}$ for all $j \neq g$.

This notation allows for an alternative notation for Equation 5:

$$k_{i,inter} \sim \frac{a_0 l}{A_i N_i} tr\left([1 - \mathbf{I}]\mathbf{N_i} H_i \mathbf{N_i}\right) \tag{7}$$

where $\mathbf{N_i}$ is the diagonal matrix of group sizes and I is the identity matrix. In other words, between-group interactions are given by the sum of off-diagonal elements of $\mathbf{N_i} H \mathbf{N_i}$.

Inter-group interactions with two groups

In the case of two groups, the inter-group term of Equation 3 of the main text becomes:

$$k_{i,inter} \sim N_i^{\delta}[2 \cdot \frac{N_{1,i}}{N_i}\frac{N_{2,i}}{N_i} - \frac{N_{1,i}}{N_i}\frac{N_{2,i}}{N_i} h^{bet}_{1,i} - \frac{N_{1,i}}{N_i}\frac{N_{2,i}}{N_i} h^{bet}_{2,i}] \tag{8}$$

Replacing $N_{2,i}$ with $N_i - N_{1,i}$ and simplifying results in:

$$k_{i,inter} \sim N^{\delta}[(\frac{N_{1,i}}{N_i}\frac{N_i - N_{1,i}}{N_i}) \cdot (2 - h^{bet}_{1,i} - h^{bet}_{2,i})] \tag{9}$$

which simplifies directly to Equation 3 of the main text when using a power-law learning curve to couple inter-croup interactions to implicit bias levels.

A Measure of Relative Mixing

Here, we compare our model in the main text to one which uses a measure of relative mixing. We start with the ratio:

$$k_{inter}/k_{within} \sim \frac{(\frac{N_1}{N} - (\frac{N_1}{N})^2)(2 - h^{bet}_1 - h^{bet}_2)}{(\frac{N_1}{N})^2(1 + h^{in}_1) + (\frac{N_2}{N})^2(1 + h^{in}_2)} \tag{10}$$

If we let the homophily and segregation terms be zero, for the moment:

$$k_{inter}/k_{within} \sim \frac{(\frac{N_1}{N} - (\frac{N_1}{N})^2)}{((\frac{N_1}{N})^2 + (\frac{N-N_1}{N})^2} \tag{11}$$

where we have substituted $N_2 = N - N_1$. Expanding out the terms in the denominator and substituting $d = \frac{N_1}{N} - (\frac{N_1}{N})^2$, with $d \in [0, .25]$, we can write:

$$k_{inter}/k_{within} \sim \frac{d}{1 - 2d} \tag{12}$$

We can directly compare this measure with the diversity part of k_{inter}, i.e. d by looking at the variance explained and information criteria of the two linear models, $ln(b) \sim ln\left(\frac{d}{1-2d}\right)$ and

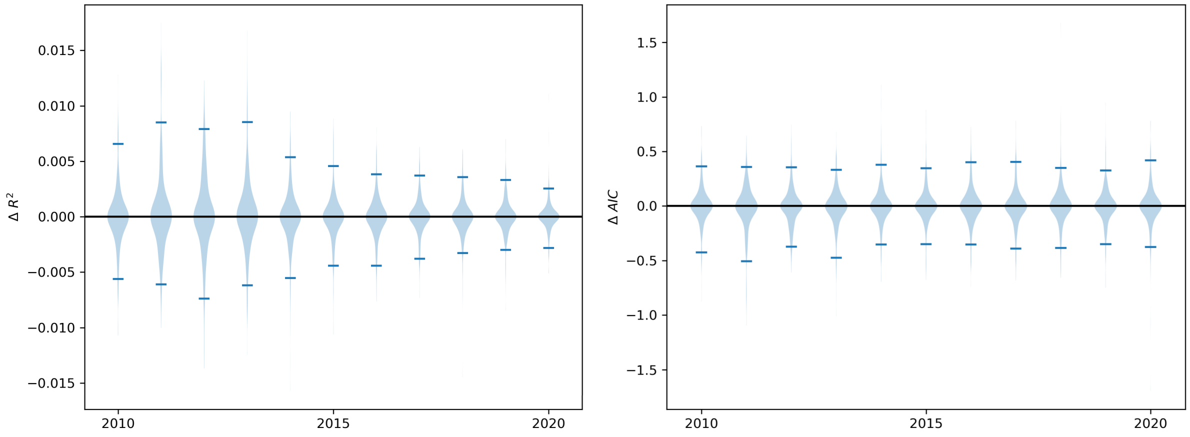

Supplementary Figure 1: The difference in R^2 (left) and AIC (right) for the two linear regressions $ln(b) \sim ln(k_{inter})$ and $ln(b) \sim ln(k_{inter}/k_{within})$. Distributions for these differences were computed via n=500 bootstrap resamples with replacement. Horizontal lines show 95% bootstrap confidence intervals.

$ln(b) \sim ln(d)$.

When comparing the R^2 and Akaike information criterion (AIC) across 500 bootstrap resamples, we find no significant advantage for either model (Supplementary Figure 1). The similarity between these two models is due to the fact that, in the absence of segregation or

homophily, k_{inter}/k_{within} is a monotonic transformation of k_{inter}. In other words, k_{inter}/k_{within} and k_{inter} necessarily are perfectly rank order correlated (i.e., $r_s = 1$).

In the presence of homophily and segregation the linear regression to predict bias becomes:

$$ln(b) \sim ln(d) + ln(2 - h_1^{bet} - h_2^{bet}) - ln\left((\frac{N_1}{N})^2(h_1^{in} - h_2^{in}) + (1 - 2\frac{N_1}{N})(1 + h_2^{in})\right) \quad (13)$$

When comparing this model to that used in the main text ($ln(b) \sim ln(d)+ln(2-h_1^{bet}-h_2^{bet})$, population term excluded) we again find no significant advantage for either model. However, there is a clear trend towards the model used in the main text, i.e., $ln(b) \sim ln(k_{inter})$ having higher adjusted R^2 and lower AIC (Supplementary Figure 2).

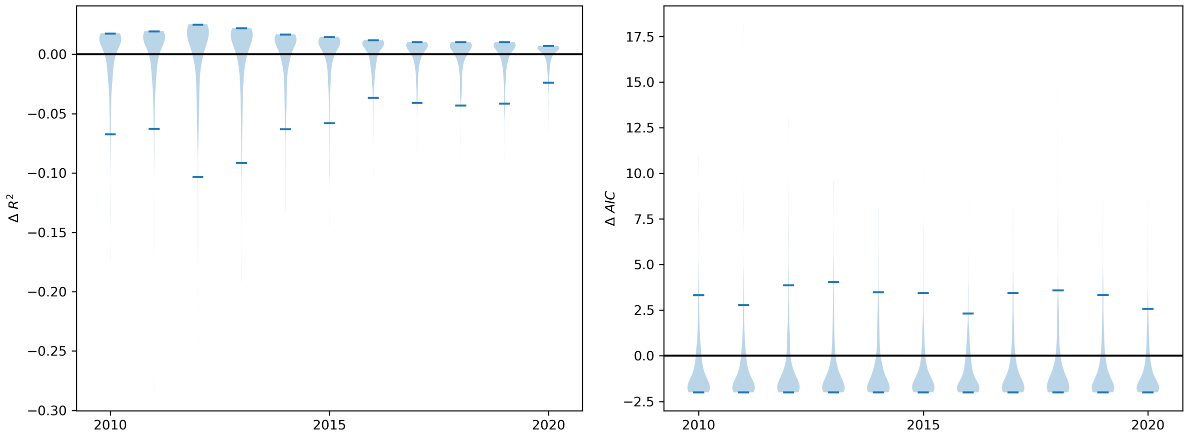

Supplementary Figure 2: The difference in R^2 (left) and AIC (right) for the two linear regressions $ln(b) \sim ln(k_{inter})$ and $ln(b) \sim ln(k_{inter}/k_{within})$ including segregation and homophily effects. Distributions for these differences were computed via n=500 bootstrap resamples with replacement. Horizontal lines show 95% bootstrap confidence intervals.

References

[1] Andrew J. Stier, Sina Sajjadi, Luıs M. A. Bettencourt, Fariba Karimi, and Marc G. Berman. Effects of racial segregation on economic productivity in u.s. cities. *arXiv*, 2022.

[2] Luís MA Bettencourt. The origins of scaling in cities. *Science*, 340(6139):1438–1441, 2013.

[3] Luís MA Bettencourt. *Introduction to urban science: evidence and theory of cities as complex systems*. MIT Press, 2021.

Supplementary Figures

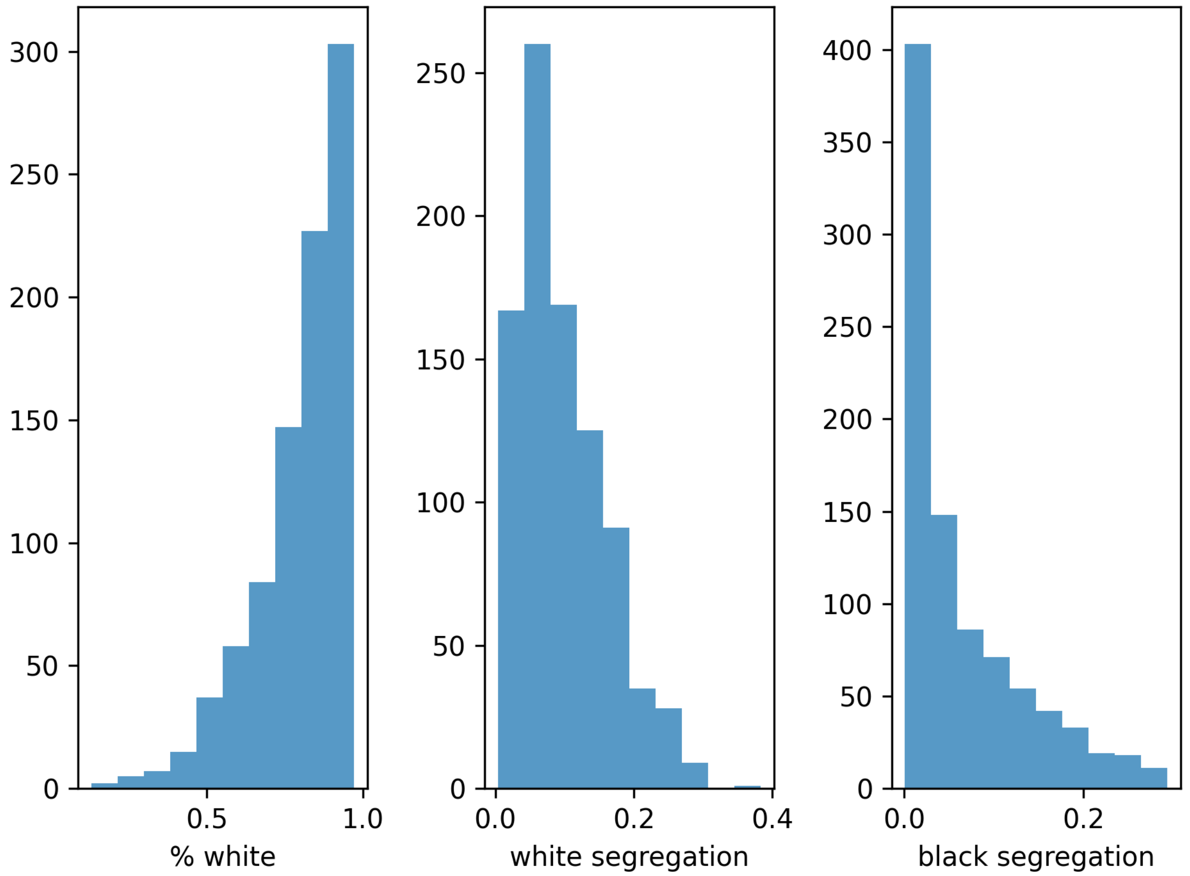

Supplementary Figure 3: Histograms of percent White, White residential racial segregation, and Black residential racial segregation for n=885 CBSAs in 2020. The y-axis denotes the number of cities in a given histogram bin.

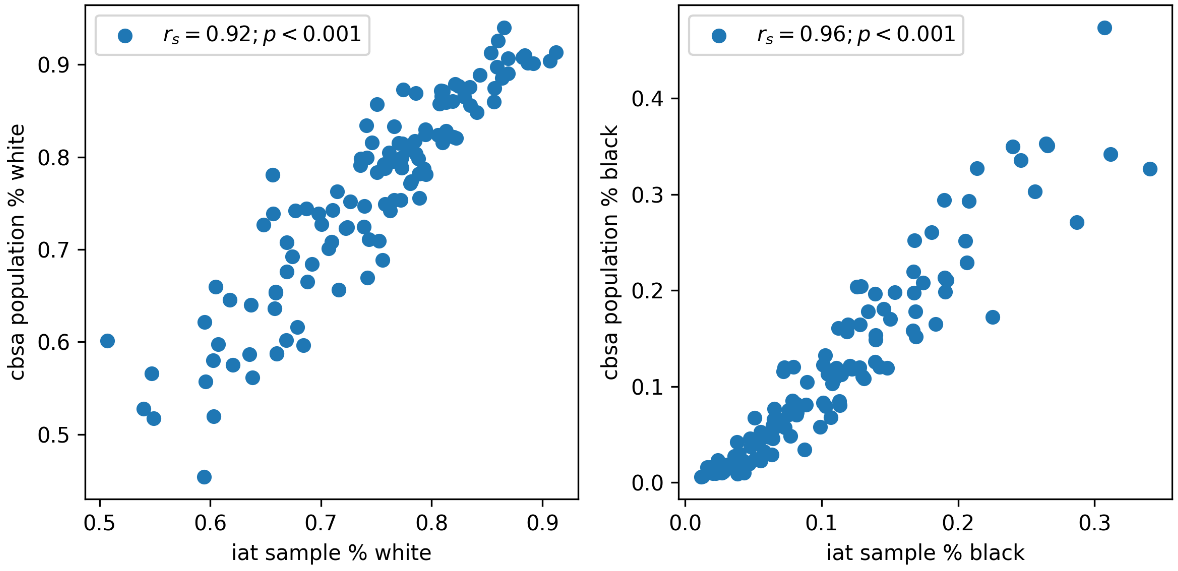

Supplementary Figure 4: Census and IAT Demographics and strongly correlated. Data for n=119 cities in 2020 demonstrates the strong correlation between the fraction of city populations that indicated their race as White or Black in the U.S. census data and the IAT sample data. Note that while the x and y axis scales are similar for the fraction of the population identified as White, the IAT sample has fewer Black respondents than would be expected from a representative sample. Nonetheless, the strong correlations indicate that the IAT sample is suitable for relative, between city analyses.

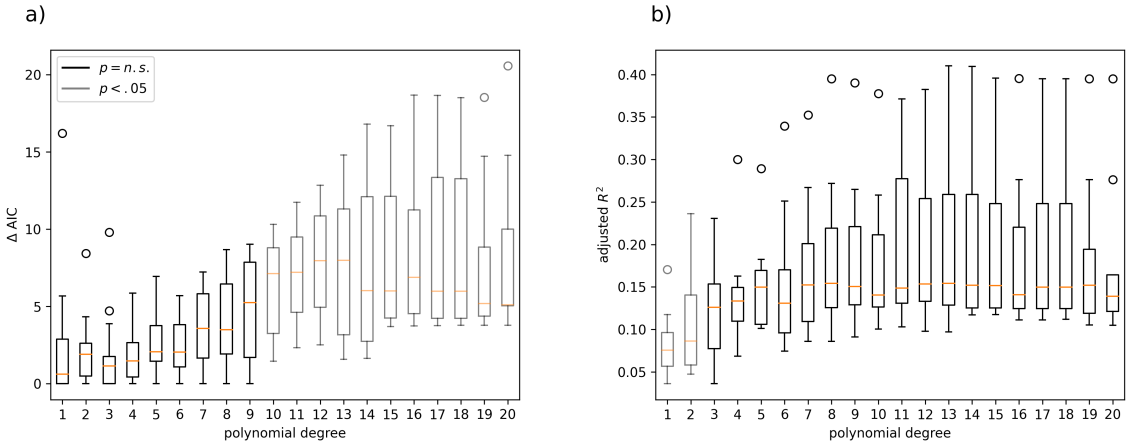

Supplementary Figure 5: Comparison of higher order coupling between h^{bet} and segregation indices. Statistically, a 4th degree polynomial is the best choice with respect to minimizing overfitting, maximizing variance explained, and having the fewest parameters. A 4th degree polynomial improves the estimated variance explained by the full model with all three effects to an average of 40% (72% of the noise ceiling median lower bound) compared to 35% (63% of the noise ceiling median lower bound) for a linear coupling (degree 1). a) That the model with a 1-degree polynomial, i.e. the linear mode used in the main text has the lowest average delta-AIC. This means that across all years and choices of segregation measure, the linear model is the least overfit model or close to as good as the least overfit model (i.e., the linear model AIC is lowest or slightly larger than the lowest AIC). The distributions of delta-AIC are not significantly different from the linear model until a degree of 10 (solid v.s. transparent). b) The adjusted coefficient of determination (R^2) is significantly larger (transparent v.s. solid) for quartic degree and higher polynomials. This difference is on the order of 5% and no further benefit to R^2 is observed for degrees beyond 4. Data for 173 total models are presented with marked medians and error bars representing the $\pm$ 1.5 inter-quartile range. Models are marked as significant based on a two-sided T-test with the degree-1 models.

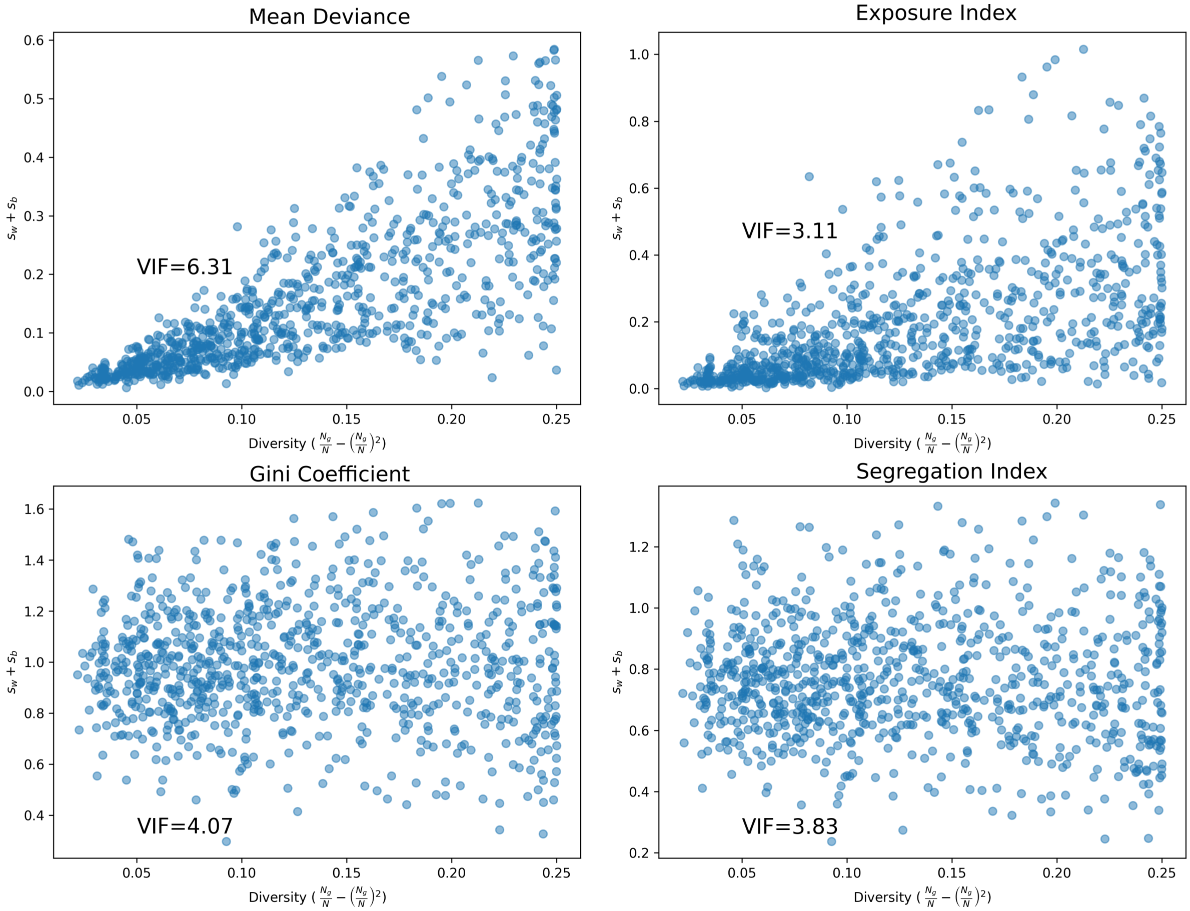

Supplementary Figure 6: Variance Inflation Factors (VIF) between the diversity and segregation term of the model are low for n=885 cities in 2020. Across all segregation measures, with the exception of the mean deviance measure, the estimated VIF is below 5. This indicates that colinearity between the diversity measure and the segregation measures is not high enough to prevent the regression model from estimating separate, independent effects. In other words, diversity and segregation effects are statistically separable even though they are correlated for some pairs of measures.

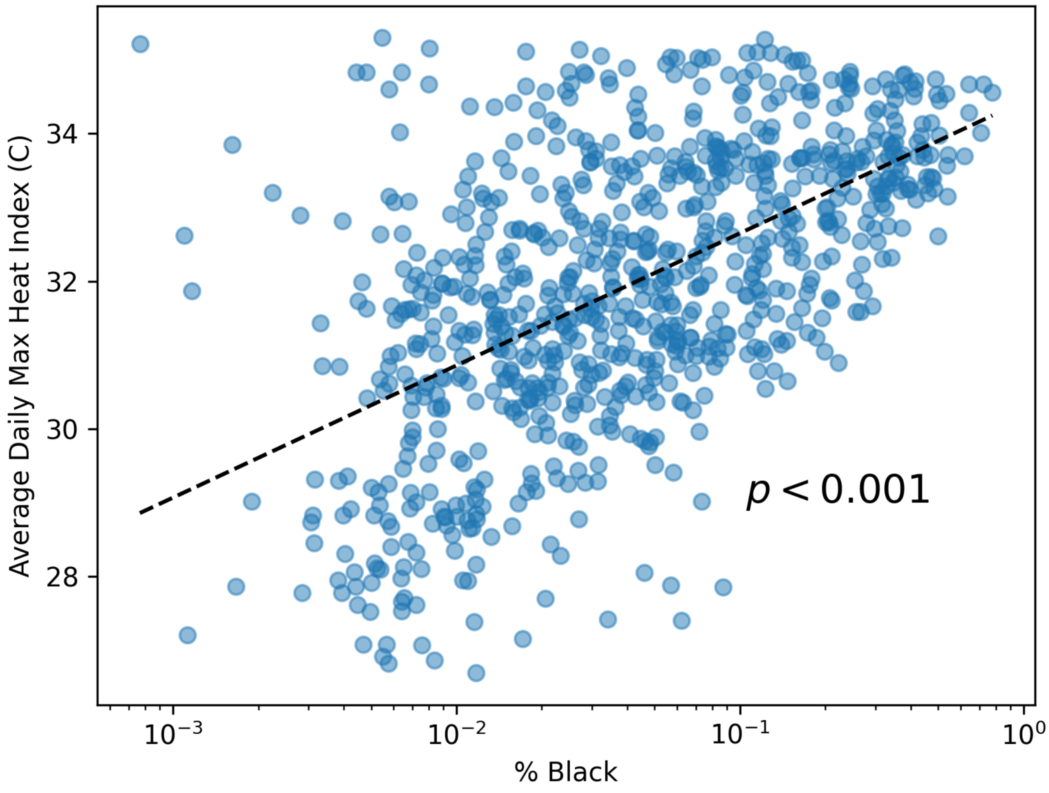

Supplementary Figure 7: Max Heat Index and racial demographics are correlated. Using data from n=314 cities in 2019 we demonstrate that the Average Daily Maximum Heat Index is significantly correlated to the percent of city populations that are Black. Combined with the analyses in Supplementary Tables 15-18, this suggests that the correlation between heat and implicit bias levels is driven by the relationship between heat and diversity, which may, in turn, be due to the historical difference in slavery, crops grown (e.g., cotton), and politics between northern and southern states.

Supplementary Tables

Supplementary Table 1: Spearman rank order correlations between empirical population fractions and IAT sample fractions across cities. Correlations across cities between e.g., the faction of the population with race reported as Black in the U.S. census data and the fraction of the city's IAT sample which self reported their race as Black. While the IAT sample is not nationally representative, these correlations suggest that the IAT sample does capture relative demographic differences between cities with respect to race.

year	r_s (White)	p value	r_s (Black)	p value	# cbsas
2010	0.91	< 0.001	0.93	< 0.001	64
2011	0.91	< 0.001	0.94	< 0.001	67
2012	0.92	< 0.001	0.94	< 0.001	67
2013	0.93	< 0.001	0.93	< 0.001	68
2014	0.90	< 0.001	0.93	< 0.001	76
2015	0.90	< 0.001	0.94	< 0.001	85
2016	0.83	< 0.001	0.93	< 0.001	91
2017	0.89	< 0.001	0.95	< 0.001	107
2018	0.90	< 0.001	0.96	< 0.001	111
2019	0.90	< 0.001	0.96	< 0.001	119
2020	0.92	< 0.001	0.96	< 0.001	119

Supplementary Table 2: Summary of scaling fits and diversity adjustment and segregation adjustment parameters for cities with more than 500 IAT responses.

year	scaling β_1	diversity adjustment β_2	$\mathbf{s_1} + \mathbf{s_2}$ β_3	# cities
2010	[-0.094,-0.021]	[-0.277,0.038]	[-0.563,0.128]	64
2011	[-0.086,-0.010]	[-0.296,0.048]	[-0.526,0.201]	59
2012	[-0.080,-0.013]	[-0.334,-0.078]	[-0.200,0.315]	46
2013	[-0.076,-0.019]	[-0.327,-0.064]	[-0.174,0.361]	52
2014	[-0.069,-0.016]	[-0.221,0.027]	[-0.321,0.225]	66
2015	[-0.047,-0.003]	[-0.305,-0.130]	[0.054,0.447]	74
2016	[-0.055,-0.015]	[-0.264,-0.095]	[0.032,0.415]	91
2017	[-0.059,-0.016]	[-0.308,-0.128]	[0.055,0.498]	105
2018	[-0.061,-0.019]	[-0.348,-0.158]	[0.105,0.563]	102
2019	[-0.063,-0.017]	[-0.292,-0.094]	[-0.073,0.409]	106
2020	[-0.044,-0.011]	[-0.278,-0.133]	[0.105,0.466]	149
all years	[-0.045,-0.031]	[-0.226,-0.163]	[0.026,0.066]	914

Supplementary Table 3: IAT participants with geographic information

year	IAT sample size	median CBSA population %
2010	163,289	0.09%
2011	149,059	0.08%
2012	118,179	0.06%
2013	125,083	0.07%
2014	171,039	0.09%
2015	208,521	0.11%
2016	273,995	0.14%
2017	321,082	0.17%
2018	316,393	0.17%
2019	321,247	0.16%
2020	584,902	0.26%

Supplementary Table 4: Logistic Regression to predict individual racial IAT bias scores $>$ 0 for 2010. Note that larger and less segregated cities are associated with a lower probability of a positive bias towards White faces, in line with Equation 3 of the main text. More diverse cities are significantly related to lower bias levels here.

**Dep. Variable:**	bias	**No. Observations:**	105199
**Model:**	Logit	**Df Residuals:**	105189
**Method:**	MLE	**Df Model:**	9
		**Pseudo R-squ.:**	0.08642
		**Log-Likelihood:**	-52444.
**converged:**	True	**LL-Null:**	-57405.
**Covariance Type:**	nonrobust	**LLR p-value:**	¡0.001

	**coef**	**std err**	**z**	**P> |z|**	**[0.025**	**0.975]**
**const**	0.9875	1.58e+05	6.25e-06	1.000	-3.1e+05	3.1e+05
**ln(population)**	-0.0174	0.010	-1.822	0.068	-0.036	0.001
**White**	0.4774	0.024	19.738	$<$ 0.001	0.430	0.525
**Black**	-1.3557	0.027	-50.526	$<$ 0.001	-1.408	-1.303
**Multiracial**	-0.1454	0.040	-3.664	$<$ 0.001	-0.223	-0.068
**High School or Less**	0.2741	1.58e+05	1.73e-06	1.000	-3.1e+05	3.1e+05
**College**	0.3079	1.58e+05	1.95e-06	1.000	-3.1e+05	3.1e+05
**Advanced Degree**	0.4055	1.58e+05	2.57e-06	1.000	-3.1e+05	3.1e+05
**Diversity**	0.0156	0.049	0.321	0.749	-0.080	0.111
**Segregation**	0.2357	0.088	2.685	0.007	0.064	0.408

Supplementary Table 5: Logistic Regression to predict individual racial IAT bias scores > 0 for 2011. Note that larger and less segregated cities are associated with a lower probability of a positive bias towards White faces, in line with Equation 3 of the main text. More diverse cities are significantly related to lower bias levels here.

**Dep. Variable:**	bias	**No. Observations:**	201378
**Model:**	Logit	**Df Residuals:**	201369
**Method:**	MLE	**Df Model:**	8
		**Pseudo R-squ.:**	0.08306
		**Log-Likelihood:**	-1.0053e+05
**converged:**	True	**LL-Null:**	-1.0963e+05
**Covariance Type:**	nonrobust	**LLR p-value:**	< 0.001

	**coef**	**std err**	**z**	**P> |z|**	**[0.025**	**0.975]**
**const**	1.2561	0.148	8.468	< 0.001	0.965	1.547
**ln(population)**	-0.0166	0.007	-2.400	0.016	-0.030	-0.003
**White**	0.4813	0.017	27.860	< 0.001	0.447	0.515
**Black**	-1.3237	0.019	-68.833	< 0.001	-1.361	-1.286
**Multiracial**	-0.1446	0.028	-5.095	< 0.001	-0.200	-0.089
**College**	0.0359	0.043	0.826	0.409	-0.049	0.121
**Advanced Degree**	0.1496	0.028	5.382	< 0.001	0.095	0.204
**Diversity**	0.0277	0.035	0.785	0.433	-0.041	0.097
**Segregation**	0.2117	0.064	3.332	0.001	0.087	0.336

Supplementary Table 6: Logistic Regression to predict individual racial IAT bias scores > 0 for 2012. Note that larger and less segregated cities are associated with a lower probability of a positive bias towards White faces, in line with Equation 3 of the main text. More diverse cities are significantly related to lower bias levels here.

**Dep. Variable:**	bias	**No. Observations:**	273788
**Model:**	Logit	**Df Residuals:**	273779
**Method:**	MLE	**Df Model:**	8
		**Pseudo R-squ.:**	0.08058
		**Log-Likelihood:**	-1.3665e+05
**converged:**	True	**LL-Null:**	-1.4863e+05
**Covariance Type:**	nonrobust	**LLR p-value:**	< 0.001

	**coef**	**std err**	**z**	**P> |z|**	**[0.025**	**0.975]**
**const**	1.1191	0.130	8.583	< 0.001	0.864	1.375
**ln(population)**	-0.0119	0.006	-1.949	0.051	-0.024	6.66e-05
**White**	0.4793	0.015	32.648	< 0.001	0.451	0.508
**Black**	-1.3083	0.016	-79.633	< 0.001	-1.341	-1.276
**Multiracial**	-0.1474	0.024	-6.080	< 0.001	-0.195	-0.100
**College**	0.0711	0.036	1.955	0.051	-0.000	0.142
**Advanced Degree**	0.1620	0.023	6.983	< 0.001	0.117	0.207
**Diversity**	0.0002	0.031	0.008	0.994	-0.060	0.060
**Segregation**	0.2315	0.054	4.260	< 0.001	0.125	0.338

Supplementary Table 7: Logistic Regression to predict individual racial IAT bias scores > 0 for 2013. Note that larger and less segregated cities are associated with a lower probability of a positive bias towards White faces, in line with Equation 3 of the main text. More diverse cities are significantly related to lower bias levels here.

**Dep. Variable:**	bias	**No. Observations:**	354724
**Model:**	Logit	**Df Residuals:**	354715
**Method:**	MLE	**Df Model:**	8
		**Pseudo R-squ.:**	0.07731
		**Log-Likelihood:**	-1.7756e+05
**converged:**	True	**LL-Null:**	-1.9243e+05
**Covariance Type:**	nonrobust	**LLR p-value:**	< 0.001

	**coef**	**std err**	**z**	**P> |z|**	**[0.025**	**0.975]**
**const**	1.1609	0.116	10.022	< 0.001	0.934	1.388
**ln(population)**	-0.0152	0.005	-2.825	0.005	-0.026	-0.005
**White**	0.4653	0.013	36.510	< 0.001	0.440	0.490
**Black**	-1.2982	0.014	-90.431	< 0.001	-1.326	-1.270
**Multiracial**	-0.1634	0.021	-7.729	< 0.001	-0.205	-0.122
**College**	0.1011	0.032	3.181	0.001	0.039	0.163
**Advanced Degree**	0.1783	0.020	9.049	< 0.001	0.140	0.217
**Diversity**	0.0046	0.027	0.167	0.867	-0.049	0.058
**Segregation**	0.2390	0.048	5.030	< 0.001	0.146	0.332

Supplementary Table 8: Logistic Regression to predict individual racial IAT bias scores > 0 for 2014. Note that larger and less segregated cities are associated with a lower probability of a positive bias towards White faces, in line with Equation 3 of the main text. More diverse cities are significantly related to lower bias levels here.

**Dep. Variable:**	bias	**No. Observations:**	476532
**Model:**	Logit	**Df Residuals:**	476523
**Method:**	MLE	**Df Model:**	8
		**Pseudo R-squ.:**	0.07018
		**Log-Likelihood:**	-2.4217e+05
**converged:**	True	**LL-Null:**	-2.6045e+05
**Covariance Type:**	nonrobust	**LLR p-value:**	< 0.001

	**coef**	**std err**	**z**	**P> |z|**	**[0.025**	**0.975]**
**const**	1.0019	0.097	10.355	< 0.001	0.812	1.191
**ln(population)**	-0.0128	0.005	-2.841	0.004	-0.022	-0.004
**White**	0.4405	0.011	40.573	< 0.001	0.419	0.462
**Black**	-1.2677	0.012	-101.960	< 0.001	-1.292	-1.243
**Multiracial**	-0.1583	0.018	-8.701	< 0.001	-0.194	-0.123
**College**	0.0879	0.026	3.372	0.001	0.037	0.139
**Advanced Degree**	0.1925	0.016	12.361	< 0.001	0.162	0.223
**Diversity**	-0.0346	0.023	-1.497	0.134	-0.080	0.011
**Segregation**	0.2801	0.041	6.905	< 0.001	0.201	0.360

Supplementary Table 9: Logistic Regression to predict individual racial IAT bias scores > 0 for 2015. Note that larger and less segregated cities are associated with a lower probability of a positive bias towards White faces, in line with Equation 3 of the main text. More diverse cities are trending in the direction of less bias, but are not significant here.

**Dep. Variable:**	bias	**No. Observations:**	507597
**Model:**	Logit	**Df Residuals:**	507588
**Method:**	MLE	**Df Model:**	8
		**Pseudo R-squ.:**	0.06866
		**Log-Likelihood:**	-2.5901e+05
**converged:**	True	**LL-Null:**	-2.7811e+05
**Covariance Type:**	nonrobust	**LLR p-value:**	< 0.001

	**coef**	**std err**	**z**	**P> |z|**	**[0.025**	**0.975]**
**const**	0.9372	0.093	10.086	< 0.001	0.755	1.119
**ln(population)**	-0.0115	0.004	-2.645	0.008	-0.020	-0.003
**White**	0.4375	0.010	41.689	< 0.001	0.417	0.458
**Black**	-1.2580	0.012	-104.296	< 0.001	-1.282	-1.234
**Multiracial**	-0.1592	0.018	-9.044	< 0.001	-0.194	-0.125
**College**	0.0931	0.025	3.727	< 0.001	0.044	0.142
**Advanced Degree**	0.1948	0.015	13.103	< 0.001	0.166	0.224
**Diversity**	-0.0500	0.022	-2.249	0.025	-0.094	-0.006
**Segregation**	0.2967	0.039	7.588	< 0.001	0.220	0.373

Supplementary Table 10: Logistic Regression to predict individual racial IAT bias scores > 0 for 2016. Note that larger, more diverse, and less segregated cities are associated with a lower probability of a positive bias towards White faces, in line with Equation 3 of the main text.

**Dep. Variable:**	bias	**No. Observations:**	77997
**Model:**	Logit	**Df Residuals:**	77987
**Method:**	MLE	**Df Model:**	9
		**Pseudo R-squ.:**	0.05309
		**Log-Likelihood:**	-39891.
**converged:**	True	**LL-Null:**	-42128.
**Covariance Type:**	nonrobust	**LLR p-value:**	< 0.001

	**coef**	**std err**	**z**	**P$>$ $\|z\|$**	**[0.025**	**0.975]**
**const**	0.9807	0.201	4.869	< 0.001	0.586	1.376
**ln(population)**	-0.0393	0.010	-3.998	< 0.001	-0.059	-0.020
**White**	0.4230	0.028	15.053	< 0.001	0.368	0.478
**Black**	-1.0896	0.033	-33.337	< 0.001	-1.154	-1.026
**Multiracial**	-0.3113	0.042	-7.336	< 0.001	-0.395	-0.228
**Birth Sex**	0.1156	0.018	6.333	< 0.001	0.080	0.151
**College**	0.2359	0.053	4.415	< 0.001	0.131	0.341
**Advanced Degree**	0.2346	0.034	6.991	< 0.001	0.169	0.300
**Diversity**	-0.1352	0.053	-2.546	0.011	-0.239	-0.031
**Segregation**	0.7152	0.102	7.035	< 0.001	0.516	0.914

Supplementary Table 11: Logistic Regression to predict individual racial IAT bias scores > 0 for 2017. Note that larger, more diverse, and less segregated cities are associated with a lower probability of a positive bias towards White faces, in line with Equation 3 of the main text.

**Dep. Variable:**	bias	**No. Observations:**	323355
**Model:**	Logit	**Df Residuals:**	323344
**Method:**	MLE	**Df Model:**	10
		**Pseudo R-squ.:**	0.04993
		**Log-Likelihood:**	-1.6958e+05
**converged:**	True	**LL-Null:**	-1.7849e+05
**Covariance Type:**	nonrobust	**LLR p-value:**	< 0.001

	**coef**	**std err**	**z**	**P> |z|**	**[0.025**	**0.975]**
**const**	0.5926	1.01e+05	5.85e-06	1.000	-1.99e+05	1.99e+05
**ln(population)**	-0.0192	0.005	-3.939	< 0.001	-0.029	-0.010
**White**	0.3823	0.014	28.240	< 0.001	0.356	0.409
**Black**	-1.1001	0.016	-68.881	< 0.001	-1.131	-1.069
**Multiracial**	-0.3696	0.020	-18.407	< 0.001	-0.409	-0.330
**Birth Sex**	0.1474	0.009	16.623	< 0.001	0.130	0.165
**High School or Less**	0.1030	1.01e+05	1.02e-06	1.000	-1.99e+05	1.99e+05
**College**	0.2474	1.01e+05	2.44e-06	1.000	-1.99e+05	1.99e+05
**Advanced Degree**	0.2422	1.01e+05	2.39e-06	1.000	-1.99e+05	1.99e+05
**Diversity**	-0.1736	0.026	-6.714	< 0.001	-0.224	-0.123
**Segregation**	0.6174	0.049	12.702	< 0.001	0.522	0.713

Supplementary Table 12: Logistic Regression to predict individual racial IAT bias scores > 0 for 2018. Note that larger, more diverse, and less segregated cities are associated with a lower probability of a positive bias towards White faces, in line with Equation 3 of the main text.

**Dep. Variable:**	bias	**No. Observations:**	563503
**Model:**	Logit	**Df Residuals:**	563493
**Method:**	MLE	**Df Model:**	9
		**Pseudo R-squ.:**	0.05051
		**Log-Likelihood:**	-2.9968e+05
**converged:**	True	**LL-Null:**	-3.1562e+05
**Covariance Type:**	nonrobust	**LLR p-value:**	< 0.001

	**coef**	**std err**	**z**	**P> |z|**	**[0.025**	**0.975]**
**const**	0.4491	0.078	5.763	< 0.001	0.296	0.602
**ln(population)**	-0.0114	0.004	-3.084	0.002	-0.019	-0.004
**White**	0.4094	0.010	40.820	< 0.001	0.390	0.429
**Black**	-1.0722	0.012	-90.542	< 0.001	-1.095	-1.049
**Multiracial**	-0.3338	0.015	-22.260	< 0.001	-0.363	-0.304
**Birth Sex**	0.1422	0.007	21.292	< 0.001	0.129	0.155
**College**	0.1656	0.020	8.453	< 0.001	0.127	0.204
**Advanced Degree**	0.1413	0.013	10.887	< 0.001	0.116	0.167
**Diversity**	-0.2113	0.020	-10.807	< 0.001	-0.250	-0.173
**Segregation**	0.6475	0.036	17.748	< 0.001	0.576	0.719

Supplementary Table 13: Logistic Regression to predict individual racial IAT bias scores > 0 for 2019. Note that larger, more diverse, and less segregated cities are associated with a lower probability of a positive bias towards White faces, in line with Equation 3 of the main text.

**Dep. Variable:**	bias	**No. Observations:**	806039
**Model:**	Logit	**Df Residuals:**	806029
**Method:**	MLE	**Df Model:**	9
		**Pseudo R-squ.:**	0.05142
		**Log-Likelihood:**	-4.3181e+05
**converged:**	True	**LL-Null:**	-4.5522e+05
**Covariance Type:**	nonrobust	**LLR p-value:**	< 0.001

	**coef**	**std err**	**z**	**P$>$ $\|z\|$**	**[0.025**	**0.975]**
**const**	0.4082	0.066	6.228	< 0.001	0.280	0.537
**ln(population)**	-0.0076	0.003	-2.456	0.014	-0.014	-0.002
**White**	0.4021	0.008	48.239	< 0.001	0.386	0.418
**Black**	-1.0854	0.010	-110.636	< 0.001	-1.105	-1.066
**Multiracial**	-0.3431	0.012	-27.498	< 0.001	-0.368	-0.319
**Birth Sex**	0.1403	0.006	25.204	< 0.001	0.129	0.151
**College**	0.1665	0.016	10.187	< 0.001	0.134	0.199
**Advanced Degree**	0.1366	0.011	12.565	< 0.001	0.115	0.158
**Diversity**	-0.2049	0.016	-12.462	< 0.001	-0.237	-0.173
**Segregation**	0.6221	0.030	20.489	< 0.001	0.563	0.682

Supplementary Table 14: Logistic Regression to predict individual racial IAT bias scores > 0 for 2020. Note that larger, more diverse, and less segregated cities are associated with a lower probability of a positive bias towards White faces, in line with Equation 3 of the main text.

**Dep. Variable:**	bias	**No. Observations:**	1288524
**Model:**	Logit	**Df Residuals:**	1288513
**Method:**	MLE	**Df Model:**	10
		**Pseudo R-squ.:**	0.04360
		**Log-Likelihood:**	-7.0843e+05
**converged:**	True	**LL-Null:**	-7.4073e+05
**Covariance Type:**	nonrobust	**LLR p-value:**	< 0.001

	**coef**	**std err**	**z**	**P> |z|**	**[0.025**	**0.975]**
**const**	0.2294	8.49e+04	2.7e-06	1.000	-1.66e+05	1.66e+05
**ln(population)**	-0.0029	0.002	-1.224	0.221	-0.008	0.002
**White**	0.3728	0.007	56.424	< 0.001	0.360	0.386
**Black**	-1.0520	0.008	-132.237	< 0.001	-1.068	-1.036
**Multiracial**	-0.3331	0.010	-33.042	< 0.001	-0.353	-0.313
**Birth Sex**	-0.2015	0.111	-1.814	0.070	-0.419	0.016
**High School or Less**	-0.0027	8.49e+04	-3.24e-08	1.000	-1.66e+05	1.66e+05
**College**	0.1431	8.49e+04	1.69e-06	1.000	-1.66e+05	1.66e+05
**Advanced Degree**	0.0890	8.49e+04	1.05e-06	1.000	-1.66e+05	1.66e+05
**Diversity**	-0.2715	0.013	-21.141	< 0.001	-0.297	-0.246
**Segregation**	0.7195	0.024	30.540	< 0.001	0.673	0.766

Supplementary Table 15: Comparison of Models for cities that have available Area Deprivation Index (ADI) and Heat Index (HI) data. All models include city size, diversity and segregation effects (mean deviation segregation measure).

year	no ADI R^2	ADI R^2	ADI n	no HI R^2	HI R^2	HI n
2010	0.383	0.394	36	0.329	0.357	18
2011	0.290	0.291	34	0.258	0.265	17
2012	0.370	0.389	27	0.463	0.478	17
2013	0.346	0.347	32	0.417	0.418	17
2014	0.248	0.259	39	0.368	0.448	18
2015	0.212	0.215	42	0.518	0.579	18
2016	0.150	0.162	50	0.502	0.546	19
2017	0.145	0.145	54	0.381	0.381	20
2018	0.204	0.222	53	0.468	0.496	19
2019	0.202	0.215	58	0.416	0.431	20
2020	0.160	0.161	76	0.437	0.438	22

Supplementary Table 16: Comparison of Models for cities that have available Area Deprivation Index (ADI) and Heat Index (HI) data. All models include city size, diversity, and segregation effects (segregation index).

year	no ADI R^2	ADI R^2	ADI n	no HI R^2	HI R^2	HI n
2010	0.399	0.412	36	0.376	0.397	18
2011	0.318	0.318	34	0.256	0.262	17
2012	0.418	0.438	27	0.517	0.527	17
2013	0.414	0.414	32	0.508	0.508	17
2014	0.289	0.300	39	0.445	0.516	18
2015	0.332	0.334	42	0.585	0.616	18
2016	0.213	0.231	50	0.639	0.662	19
2017	0.193	0.193	54	0.559	0.562	20
2018	0.273	0.293	53	0.671	0.690	19
2019	0.293	0.310	58	0.656	0.664	20
2020	0.288	0.288	76	0.650	0.650	22

Supplementary Table 17: Comparison of Models for cities that have available Area Deprivation Index (ADI) and Heat Index (HI) data. All models include city size, diversity and segregation effects (gini coefficient).

year	no ADI R^2	ADI R^2	ADI n	no HI R^2	HI R^2	HI n
2010	0.401	0.416	36	0.366	0.388	18
2011	0.327	0.327	34	0.257	0.263	17
2012	0.424	0.448	27	0.518	0.529	17
2013	0.414	0.415	32	0.502	0.502	17
2014	0.291	0.300	39	0.442	0.517	18
2015	0.333	0.338	42	0.581	0.617	18
2016	0.223	0.239	50	0.633	0.660	19
2017	0.197	0.197	54	0.568	0.569	20
2018	0.271	0.288	53	0.665	0.686	19
2019	0.296	0.310	58	0.648	0.658	20
2020	0.296	0.296	76	0.651	0.652	22

Supplementary Table 18: Comparison of Models for cities that have available Area Deprivation Index (ADI) and Heat Index (HI) data. All models include city size, diversity and segregation effects (η^2).

year	no ADI R^2	ADI R^2	ADI n	no HI R^2	HI R^2	HI n
2010	0.394	0.406	36	0.365	0.387	18
2011	0.310	0.310	34	0.252	0.259	17
2012	0.410	0.429	27	0.505	0.515	17
2013	0.406	0.406	32	0.490	0.490	17
2014	0.289	0.300	39	0.426	0.498	18
2015	0.293	0.296	42	0.589	0.623	18
2016	0.187	0.202	50	0.582	0.609	19
2017	0.178	0.179	54	0.494	0.497	20
2018	0.246	0.265	53	0.575	0.590	19
2019	0.258	0.270	58	0.556	0.561	20
2020	0.225	0.225	76	0.561	0.563	22

Supplementary Table 19: Summary of scaling fits and diversity adjustment and segregation adjustment variance explained for cities with more than 250 IAT responses.

year	scaling R^2	diversity adjustment R^2	segregation adjustment R^2	overall R^2	# cities
2010	0.125	0.128	-0.007	0.255	126
2011	0.056	0.120	-0.005	0.188	119
2012	0.165	0.047	-0.008	0.225	88
2013	0.134	0.091	-0.008	0.232	98
2014	0.097	0.066	-0.008	0.172	116
2015	0.044	0.158	-0.004	0.211	129
2016	0.078	0.145	0.021	0.246	148
2017	0.079	0.132	0.015	0.232	163
2018	0.143	0.131	0.061	0.323	157
2019	0.054	0.168	0.008	0.236	174
2020	0.046	0.059	0.040	0.150	228
all years	0.086	0.117	0.003	0.205	1546

Supplementary Table 20: Summary of scaling fits and diversity adjustment and segregation adjustment variance explained for cities with more than 1000 IAT responses.

year	scaling R^2	diversity adjustment R^2	segregation adjustment R^2	overall R^2	# cities
2010	0.053	0.143	-0.022	0.239	31
2011	0.112	0.145	-0.030	0.284	31
2012	0.047	0.082	-0.019	0.195	26
2013	0.052	0.195	0.016	0.319	28
2014	0.039	0.262	0.036	0.371	34
2015	0.014	0.340	0.055	0.436	43
2016	0.115	0.227	0.022	0.369	57
2017	0.071	0.207	0.045	0.339	61
2018	0.090	0.210	0.022	0.337	59
2019	0.054	0.169	-0.002	0.248	61
2020	0.041	0.147	0.094	0.292	89
all years	0.076	0.201	0.026	0.298	520

Supplementary Table 21: Summary of scaling fits and diversity adjustment and segregation adjustment variance explained from the η^2 measure for cities with more than 250 IAT responses.

year	scaling R^2	diversity adjustment R^2	segregation adjustment R^2	overall R^2	# cities
2010	0.125	0.128	0.010	0.265	126
2011	0.056	0.120	0.012	0.199	119
2012	0.165	0.047	0.002	0.228	88
2013	0.134	0.091	0.035	0.263	98
2014	0.097	0.066	0.004	0.180	116
2015	0.044	0.158	0.033	0.243	129
2016	0.078	0.145	0.050	0.270	148
2017	0.079	0.132	0.071	0.278	163
2018	0.143	0.131	0.118	0.368	157
2019	0.054	0.168	0.052	0.274	174
2020	0.046	0.059	0.094	0.199	228
all years	0.086	0.117	0.036	0.231	1546

Supplementary Table 22: Summary of scaling fits and diversity adjustment and segregation adjustment variance explained from the η^2 measure for cities with more than 1000 IAT responses.

year	scaling R^2	diversity adjustment R^2	segregation adjustment R^2	overall R^2	# cities
2010	0.053	0.143	0.024	0.276	31
2011	0.112	0.145	-0.008	0.299	31
2012	0.047	0.082	0.069	0.269	26
2013	0.052	0.195	0.115	0.401	28
2014	0.039	0.262	0.085	0.413	34
2015	0.014	0.340	0.126	0.501	43
2016	0.115	0.227	0.104	0.434	57
2017	0.071	0.207	0.125	0.407	61
2018	0.090	0.210	0.091	0.393	59
2019	0.054	0.169	0.065	0.306	61
2020	0.041	0.147	0.167	0.358	89
all years	0.076	0.201	0.088	0.351	520

Supplementary Table 23: Summary of scaling fits and diversity adjustment and segregation adjustment variance explained from the segregation index for cities with more than 250 IAT responses.

year	scaling R^2	diversity adjustment R^2	segregation adjustment R^2	overall R^2	# cities
2010	0.125	0.128	0.023	0.274	126
2011	0.056	0.120	0.024	0.210	119
2012	0.165	0.047	0.006	0.231	88
2013	0.134	0.091	0.053	0.277	98
2014	0.097	0.066	0.009	0.183	116
2015	0.044	0.158	0.045	0.253	129
2016	0.078	0.145	0.092	0.307	148
2017	0.079	0.132	0.085	0.290	163
2018	0.143	0.131	0.137	0.382	157
2019	0.054	0.168	0.103	0.320	174
2020	0.046	0.059	0.148	0.250	228
all years	0.086	0.117	0.064	0.255	1546

Supplementary Table 24: Summary of scaling fits and diversity adjustment and segregation adjustment variance explained from the segregation index for cities with more than 1000 IAT responses.

year	scaling R^2	diversity adjustment R^2	segregation adjustment R^2	overall R^2	# cities
2010	0.053	0.143	0.040	0.288	31
2011	0.112	0.145	0.004	0.307	31
2012	0.047	0.082	0.121	0.312	26
2013	0.052	0.195	0.136	0.418	28
2014	0.039	0.262	0.079	0.408	34
2015	0.014	0.340	0.134	0.508	43
2016	0.115	0.227	0.113	0.441	57
2017	0.071	0.207	0.134	0.414	61
2018	0.090	0.210	0.092	0.395	59
2019	0.054	0.169	0.085	0.324	61
2020	0.041	0.147	0.195	0.383	89
all years	0.076	0.201	0.111	0.372	520

Supplementary Table 25: Summary of scaling fits and diversity adjustment and segregation adjustment variance explained from the gini coefficeint for cities with more than 250 IAT responses.

year	scaling R^2	diversity adjustment R^2	segregation adjustment R^2	overall R^2	# cities
2010	0.125	0.128	0.031	0.281	126
2011	0.056	0.120	0.027	0.212	119
2012	0.165	0.047	0.007	0.231	88
2013	0.134	0.091	0.059	0.282	98
2014	0.097	0.066	0.009	0.184	116
2015	0.044	0.158	0.050	0.258	129
2016	0.078	0.145	0.100	0.313	148
2017	0.079	0.132	0.104	0.306	163
2018	0.143	0.131	0.147	0.391	157
2019	0.054	0.168	0.106	0.323	174
2020	0.046	0.059	0.158	0.260	228
all years	0.086	0.117	0.067	0.258	1546

Supplementary Table 26: Summary of scaling fits and diversity adjustment and segregation adjustment variance explained from the gini coefficeint for cities with more than 1000 IAT responses.

year	scaling R^2	diversity adjustment R^2	segregation adjustment R^2	overall R^2	# cities
2010	0.053	0.143	0.044	0.291	31
2011	0.112	0.145	0.010	0.310	31
2012	0.047	0.082	0.120	0.311	26
2013	0.052	0.195	0.128	0.410	28
2014	0.039	0.262	0.079	0.408	34
2015	0.014	0.340	0.118	0.494	43
2016	0.115	0.227	0.105	0.434	57
2017	0.071	0.207	0.136	0.415	61
2018	0.090	0.210	0.093	0.395	59
2019	0.054	0.169	0.078	0.317	61
2020	0.041	0.147	0.205	0.393	89
all years	0.076	0.201	0.111	0.371	520

Supplementary Table 27: Summary of scaling fits and diversity adjustment and segregation variance explained for cities with more than 500 IAT responses.

year	scaling R^2	diversity adjustment R^2	segregation adjustment R^2	overall R^2	# cities
2010	0.122	0.180	0.008	0.327	64
2011	0.086	0.134	-0.003	0.246	59
2012	0.130	0.266	-0.014	0.400	46
2013	0.164	0.194	-0.008	0.368	52
2014	0.125	0.105	-0.012	0.242	66
2015	0.053	0.216	0.055	0.336	74
2016	0.112	0.121	0.041	0.277	91
2017	0.097	0.155	0.040	0.295	105
2018	0.114	0.170	0.057	0.335	102
2019	0.094	0.147	0.007	0.257	106
2020	0.061	0.128	0.049	0.242	149
all years	0.105	0.160	0.008	0.267	914

Supplementary Table 28: Summary of scaling fits and diversity adjustment and segregation variance explained estimated from the segregation index for cities with more than 500 IAT responses.

year	scaling R^2	diversity adjustment R^2	segregation adjustment R^2	overall R^2	# cities
2011	0.086	0.134	0.001	0.245	59
2012	0.130	0.266	0.053	0.448	46
2013	0.164	0.194	0.066	0.418	52
2014	0.125	0.105	0.016	0.260	66
2015	0.053	0.216	0.169	0.436	74
2016	0.112	0.121	0.128	0.348	91
2017	0.097	0.155	0.091	0.336	105
2018	0.114	0.170	0.134	0.397	102
2019	0.094	0.147	0.083	0.318	106
2020	0.061	0.128	0.160	0.341	149
all years	0.105	0.160	0.078	0.324	914

Supplementary Table 29: Summary of scaling fits and diversity adjustment and segregation adjustment parameters estimated from the segregation index for cities with more than 500 IAT responses.

year	scaling β_1	diversity adjustment β_2	$\mathbf{s_1} + \mathbf{s_2}\ \beta_3$	# cities
2010	[-0.094,-0.021]	[-0.303,-0.096]	[-0.135,0.214]	64
2011	[-0.086,-0.010]	[-0.305,-0.073]	[-0.090,0.281]	59
2012	[-0.080,-0.013]	[-0.282,-0.107]	[0.005,0.261]	46
2013	[-0.076,-0.019]	[-0.260,-0.088]	[0.020,0.273]	52
2014	[-0.069,-0.016]	[-0.209,-0.050]	[-0.034,0.226]	66
2015	[-0.047,-0.003]	[-0.210,-0.106]	[0.114,0.290]	74
2016	[-0.055,-0.015]	[-0.174,-0.068]	[0.091,0.270]	91
2017	[-0.059,-0.016]	[-0.210,-0.098]	[0.090,0.302]	105
2018	[-0.061,-0.019]	[-0.238,-0.120]	[0.137,0.354]	102
2019	[-0.063,-0.017]	[-0.229,-0.105]	[0.091,0.331]	106
2020	[-0.044,-0.011]	[-0.183,-0.093]	[0.178,0.362]	149
all years	[-0.045,-0.031]	[-0.179,-0.139]	[0.135,0.201]	914

Supplementary Table 30: Summary of scaling fits and diversity adjustment and segregation variance explained estimated from the gini coefficient for cities with more than 500 IAT responses.

year	scaling R^2	diversity adjustment R^2	segregation adjustment R^2	overall R^2	# cities
2010	0.122	0.180	-0.009	0.309	64
2011	0.086	0.134	0.008	0.251	59
2012	0.130	0.266	0.053	0.448	46
2013	0.164	0.194	0.058	0.413	52
2014	0.125	0.105	0.018	0.261	66
2015	0.053	0.216	0.166	0.433	74
2016	0.112	0.121	0.135	0.354	91
2017	0.097	0.155	0.099	0.343	105
2018	0.114	0.170	0.143	0.405	102
2019	0.094	0.147	0.080	0.316	106
2020	0.061	0.128	0.170	0.350	149
all years	0.105	0.160	0.082	0.327	914

Supplementary Table 31: Summary of scaling fits and diversity adjustment and segregation adjustment parameters estimated from the gini coefficient for cities with more than 500 IAT responses.

year	scaling β_1	diversity adjustment β_2	$\mathbf{s_1} + \mathbf{s_2}$ β_3	# cities
2010	[-0.094,-0.021]	[-0.305,-0.097]	[-0.118,0.206]	64
2011	[-0.086,-0.010]	[-0.310,-0.077]	[-0.066,0.276]	59
2012	[-0.080,-0.013]	[-0.285,-0.110]	[0.005,0.239]	46
2013	[-0.076,-0.019]	[-0.262,-0.088]	[0.012,0.243]	52
2014	[-0.069,-0.016]	[-0.212,-0.052]	[-0.029,0.207]	66
2015	[-0.047,-0.003]	[-0.213,-0.108]	[0.099,0.256]	74
2016	[-0.055,-0.015]	[-0.177,-0.071]	[0.084,0.240]	91
2017	[-0.059,-0.016]	[-0.212,-0.100]	[0.086,0.270]	105
2018	[-0.061,-0.019]	[-0.241,-0.124]	[0.127,0.315]	102
2019	[-0.063,-0.017]	[-0.232,-0.106]	[0.076,0.283]	106
2020	[-0.044,-0.011]	[-0.187,-0.097]	[0.163,0.321]	149
all years	[-0.045,-0.031]	[-0.182,-0.142]	[0.164,0.241]	914

Supplementary Table 32: Summary of scaling fits and diversity adjustment and segregation variance explained estimated from the η^2 measure for cities with more than 500 IAT responses.

year	scaling R^2	diversity adjustment R^2	segregation adjustment R^2	overall R^2	# cities
2010	0.122	0.180	-0.010	0.309	64
2011	0.086	0.134	-0.001	0.244	59
2012	0.130	0.266	0.043	0.442	46
2013	0.164	0.194	0.062	0.418	52
2014	0.125	0.105	0.015	0.260	66
2015	0.053	0.216	0.140	0.410	74
2016	0.112	0.121	0.091	0.317	91
2017	0.097	0.155	0.097	0.341	105
2018	0.114	0.170	0.116	0.382	102
2019	0.094	0.147	0.056	0.296	106
2020	0.061	0.128	0.115	0.300	149
all years	0.105	0.160	0.056	0.305	914

Supplementary Table 33: Summary of scaling fits and diversity adjustment and segregation adjustment parameters estimated from the η^2 measure for cities with more than 500 IAT responses.

year	scaling β_1	diversity adjustment β_2	$\mathbf{s_1} + \mathbf{s_2}$ β_3	# cities
2010	[-0.094,-0.021]	[-0.334,-0.091]	[-0.105,0.177]	64
2011	[-0.086,-0.010]	[-0.348,-0.081]	[-0.077,0.224]	59
2012	[-0.080,-0.013]	[-0.329,-0.131]	[-0.004,0.204]	46
2013	[-0.076,-0.019]	[-0.314,-0.118]	[0.014,0.228]	52
2014	[-0.069,-0.016]	[-0.249,-0.059]	[-0.031,0.195]	66
2015	[-0.047,-0.003]	[-0.269,-0.142]	[0.083,0.242]	74
2016	[-0.055,-0.015]	[-0.230,-0.101]	[0.057,0.221]	91
2017	[-0.059,-0.016]	[-0.276,-0.140]	[0.086,0.277]	105
2018	[-0.061,-0.019]	[-0.309,-0.164]	[0.108,0.306]	102
2019	[-0.063,-0.017]	[-0.285,-0.130]	[0.049,0.265]	106
2020	[-0.044,-0.011]	[-0.255,-0.143]	[0.118,0.280]	149
all years	[-0.045,-0.031]	[-0.229,-0.181]	[0.050,0.077]	914

Supplementary Table 34: Comparison of noise ceiling estimates and full sample R^2 for the deviance measure of segregation a threshold of >500 responses per city.

year	noise ceiling R^2 range	Full Sample R^2	Lower Bound Noise Corrected R^2
2010	[0.70, 0.92]	0.34	0.48
2011	[0.68, 0.91]	0.26	0.38
2012	[0.53, 0.86]	0.41	0.78
2013	[0.53, 0.86]	0.38	0.72
2014	[0.54, 0.87]	0.25	0.47
2015	[0.37, 0.80]	0.34	0.93
2016	[0.45, 0.84]	0.29	0.63
2017	[0.62, 0.89]	0.30	0.49
2018	[0.60, 0.89]	0.34	0.57
2019	[0.57, 0.88]	0.26	0.47
2020	[0.45, 0.83]	0.25	0.55

Supplementary Table 35: Comparison of noise ceiling estimates and full sample R^2 for the deviance measure of segregation a threshold of >250 responses per city.

year	Full Sample R^2	Lower Bound Noise Corrected R^2
2010	0.26	0.47
2011	0.20	0.37
2012	0.23	0.54
2013	0.24	0.70
2014	0.18	0.49
2015	0.22	0.57
2016	0.25	0.69
2017	0.24	0.51
2018	0.33	0.72
2019	0.24	0.55
2020	0.15	0.45

Supplementary Table 36: Comparison of noise ceiling estimates and full sample R^2 for the deviance measure of segregation a threshold of $>$1000 responses per city.

year	Full Sample R^2	Lower Bound Noise Corrected R^2
2010	0.26	0.37
2011	0.31	0.41
2012	0.23	0.41
2013	0.34	0.65
2014	0.39	0.77
2015	0.45	0.79
2016	0.38	0.76
2017	0.35	0.55
2018	0.35	0.50
2019	0.26	0.36
2020	0.30	0.50

Supplementary Table 37: Comparison of noise ceiling estimates and full sample R^2 for the η^2 measure of segregation and a threshold of $>$500 responses per city.

year	Full Sample R^2	Lower Bound Noise Corrected R^2
2010	0.32	0.46
2011	0.26	0.38
2012	0.45	0.86
2013	0.43	0.81
2014	0.27	0.51
2015	0.42	1.13
2016	0.32	0.72
2017	0.35	0.56
2018	0.39	0.65
2019	0.30	0.53
2020	0.30	0.68

Supplementary Table 38: Comparison of noise ceiling estimates and full sample R^2 for the η^2 measure of segregation and a threshold of >250 responses per city.

year	Full Sample R^2	Lower Bound Noise Corrected R^2
2010	0.27	0.49
2011	0.21	0.39
2012	0.24	0.55
2013	0.27	0.79
2014	0.19	0.51
2015	0.25	0.65
2016	0.28	0.75
2017	0.28	0.61
2018	0.37	0.81
2019	0.28	0.64
2020	0.20	0.59

Supplementary Table 39: Comparison of noise ceiling estimates and full sample R^2 for the η^2 measure of segregation and a threshold of >1000 responses per city.

year	Full Sample R^2	Lower Bound Noise Corrected R^2
2010	0.30	0.42
2011	0.32	0.43
2012	0.30	0.54
2013	0.42	0.80
2014	0.43	0.85
2015	0.51	0.90
2016	0.44	0.88
2017	0.42	0.65
2018	0.40	0.58
2019	0.32	0.44
2020	0.37	0.61

Supplementary Table 40: Comparison of noise ceiling estimates and full sample R^2 for the gini coefficient measure of segregation and a threshold of >500 responses per city.

year	Full Sample R^2	Lower Bound Noise Corrected R^2
2010	0.32	0.46
2011	0.26	0.39
2012	0.46	0.87
2013	0.42	0.80
2014	0.27	0.51
2015	0.44	1.19
2016	0.36	0.80
2017	0.35	0.56
2018	0.41	0.68
2019	0.32	0.57
2020	0.35	0.80

Supplementary Table 41: Comparison of noise ceiling estimates and full sample R^2 for the gini coefficient measure of segregation and a threshold of >250 responses per city.

year	Full Sample R^2	Lower Bound Noise Corrected R^2
2010	0.29	0.52
2011	0.22	0.41
2012	0.24	0.55
2013	0.29	0.84
2014	0.19	0.53
2015	0.26	0.69
2016	0.32	0.87
2017	0.31	0.67
2018	0.39	0.86
2019	0.33	0.75
2020	0.26	0.77

Supplementary Table 42: Comparison of noise ceiling estimates and full sample R^2 for the gini coefficient measure of segregation and a threshold of >1000 responses per city.

year	Full Sample R^2	Lower Bound Noise Corrected R^2
2010	0.31	0.44
2011	0.33	0.45
2012	0.34	0.62
2013	0.43	0.82
2014	0.43	0.84
2015	0.51	0.89
2016	0.44	0.88
2017	0.43	0.66
2018	0.41	0.58
2019	0.33	0.46
2020	0.40	0.67

Supplementary Table 43: Comparison of noise ceiling estimates and full sample R^2 for the segregation index measure of segregation and a threshold of >500 responses per city.

year	Full Sample R^2	Lower Bound Noise Corrected R^2
2010	0.32	0.45
2011	0.26	0.38
2012	0.46	0.87
2013	0.43	0.81
2014	0.27	0.51
2015	0.44	1.20
2016	0.36	0.78
2017	0.34	0.55
2018	0.40	0.67
2019	0.32	0.57
2020	0.35	0.78

Supplementary Table 44: Comparison of noise ceiling estimates and full sample R^2 for the segregation index measure of segregation and a threshold of $>$250 responses per city.

year	Full Sample R^2	Lower Bound Noise Corrected R^2
2010	0.28	0.50
2011	0.22	0.41
2012	0.24	0.55
2013	0.28	0.83
2014	0.19	0.52
2015	0.26	0.68
2016	0.31	0.85
2017	0.29	0.64
2018	0.39	0.85
2019	0.32	0.74
2020	0.25	0.74

Supplementary Table 45: Comparison of noise ceiling estimates and full sample R^2 for the segregation index measure of segregation and a threshold of $>$1000 responses per city.

year	Full Sample R^2	Lower Bound Noise Corrected R^2
2010	0.31	0.44
2011	0.33	0.44
2012	0.34	0.62
2013	0.44	0.83
2014	0.43	0.84
2015	0.52	0.92
2016	0.45	0.90
2017	0.42	0.66
2018	0.41	0.58
2019	0.34	0.47
2020	0.39	0.65
