# Supplementary material for: Implicit racial biases are lower in more populous more diverse and less segregated US cities
Source: Nat Commun. 2024 Feb 6;15:961. doi: 10.1038/s41467-024-45013-8 (PMC10847142; doi:10.1038/s41467-024-45013-8)
Supplement: Supplementary file 3 — Description of Additional Supplementary Files [file 41467_2024_45013_MOESM3_ESM.pdf]

Description of Additional Supplementary Files

File Name: Supplementary Data 1
Description: Complete model results and statistics

File Name: Supplementary Data 2
Description: Metropolitan Areas Included by year
