# Supplementary material for: Implicit racial biases are lower in more populous more diverse and less segregated US cities
Source: Nat Commun. 2024 Feb 6;15:961. doi: 10.1038/s41467-024-45013-8 (PMC10847142; doi:10.1038/s41467-024-45013-8)
Supplement: Supplementary file 6 — Reporting Summary [file 41467_2024_45013_MOESM6_ESM.pdf]

nature portfolio

Corresponding author(s): Andrew J. Stier
Marc G. Berman

Last updated by author(s): Dec 4, 2023

Reporting Summary

Nature Portfolio wishes to improve the reproducibility of the work that we publish. This form provides structure for consistency and transparency in reporting. For further information on Nature Portfolio policies, see our Editorial Policies and the Editorial Policy Checklist.

Statistics

For all statistical analyses, confirm that the following items are present in the figure legend, table legend, main text, or Methods section.

n/a	Confirmed	
☐	☒	The exact sample size (*n*) for each experimental group/condition, given as a discrete number and unit of measurement
☒	☐	A statement on whether measurements were taken from distinct samples or whether the same sample was measured repeatedly
☐	☒	The statistical test(s) used AND whether they are one- or two-sided<br>*Only common tests should be described solely by name; describe more complex techniques in the Methods section.*
☐	☒	A description of all covariates tested
☐	☒	A description of any assumptions or corrections, such as tests of normality and adjustment for multiple comparisons
☐	☒	A full description of the statistical parameters including central tendency (e.g. means) or other basic estimates (e.g. regression coefficient) AND variation (e.g. standard deviation) or associated estimates of uncertainty (e.g. confidence intervals)
☐	☒	For null hypothesis testing, the test statistic (e.g. *F*, *t*, *r*) with confidence intervals, effect sizes, degrees of freedom and *P* value noted<br>*Give P values as exact values whenever suitable.*
☒	☐	For Bayesian analysis, information on the choice of priors and Markov chain Monte Carlo settings
☒	☐	For hierarchical and complex designs, identification of the appropriate level for tests and full reporting of outcomes
☐	☒	Estimates of effect sizes (e.g. Cohen's *d*, Pearson's *r*), indicating how they were calculated

Our web collection on statistics for biologists contains articles on many of the points above.

Software and code

Policy information about availability of computer code

Data collection	No software was used
Data analysis	This analysis software was developed using python 3.11 and the following package versions: numpy 1.24.3 pandas 1.5.3 matplotlib 3.7.1 statsmodels 0.14.1 scipy 1.10.1. All data analysis was conducted in python and analysis code is available at https://github.com/enlberman/implicit_biases_cities (DOI: 10.5281/zenodo.10258104).

For manuscripts utilizing custom algorithms or software that are central to the research but not yet described in published literature, software must be made available to editors and reviewers. We strongly encourage code deposition in a community repository (e.g. GitHub). See the Nature Portfolio guidelines for submitting code & software for further information.

Data

Policy information about availability of data

All manuscripts must include a data availability statement. This statement should provide the following information, where applicable:

- Accession codes, unique identifiers, or web links for publicly available datasets
- A description of any restrictions on data availability
- For clinical datasets or third party data, please ensure that the statement adheres to our policy

All data needed to evaluate the conclusions in the paper are present in the paper, the Supplementary Materials, or are publicly available. Source data are provided with this paper. IAT data can be found at: https://osf.io/52qxl/. U.S. Census data can be found at: https://data.census.gov. CBSA delineation files can be found at:

https://www.census.gov/programs-surveys/metro-micro/about/delineation-files.html. ADI data can be found at: https://www.neighborhoodatlas.medicine.wisc.edu/. North America Land Data Assimilation System Daily Air Temperatures and Heat Index data can be found at: https://wonder.cdc.gov/nasa-nldas.html.

Research involving human participants, their data, or biological material

Policy information about studies with human participants or human data. See also policy information about sex, gender (identity/presentation), and sexual orientation and race, ethnicity and racism.

Reporting on sex and gender	Birth sex was available by self report for 1,549,567 of the study participants. In individual level logistic regressions we found that individuals who indicated their birth sex as Male tended to have higher implicit biases, but that this was independent of city population, diversity, and segregation effects, suggesting that the results of the study are robust to sex. Of the participants who reported sex information 579,554 were male and 970,013 were female.
Reporting on race, ethnicity, or other socially relevant groupings	To control for potential individual level confounders we incorporated analyses in which we put the effects from our model (city population, city diversity, and city segregation) and self reported race/ethnicity and education into a logistic regression. race/ethnicity were collapsed into three categories of other, white, black, and multiracial. This choice was made due to the fact that the racial IAT data we analyzed specifically contrasts black and white faces. Education was collapsed into categories of "less than college/other", "College degree", and "Advanced degree".
Population characteristics	See above
Recruitment	Data were publicly available and no participants were recruited specifically for this study.
Ethics oversight	Collection of IAT data via the project implicit website and relevant consent procedures are overseen by the University of Virginia Institutional Review Board for the Social and Behavioral Sciences. Additionally, the merging of IAT data with US Census data was approved by the University of Chicago Institutional Review Board under IRB23-0796.

Note that full information on the approval of the study protocol must also be provided in the manuscript.

Field-specific reporting

Please select the one below that is the best fit for your research. If you are not sure, read the appropriate sections before making your selection.

☐ Life sciences ☒ Behavioural & social sciences ☐ Ecological, evolutionary & environmental sciences

For a reference copy of the document with all sections, see nature.com/documents/nr-reporting-summary-flat.pdf

Behavioural & social sciences study design

All studies must disclose on these points even when the disclosure is negative.

Study description	This quantitative study uses publicly available data from the project implicit racial IAT to test a model that explain variation in implicit racial biases with city population, diversity, and segregation.
Research sample	The research sample includes all individuals who completed a online racial IAT between 2010 and 2020 and provided geographic information. These online implicit bias tests are hosted by Harvard's Project Implicit at https://implicit.harvard.edu/implicit/takeatest.html. This is a convenience sample of publicly available data and was not collected specifically for this manuscript. This sample is not representative of the U.S. population. IAT data can be found at: https://osf.io/52qxl/.
Sampling strategy	There was no sampling. We used all available data from the project implicit racial IAT.
Data collection	Data was collected via participation on the project implicit website. Computers or Mobile phones can be used to access the website. The researchers who designed the data collection website did so with no knowledge of the current study.
Timing	Data were collected between 2010 and 2020.
Data exclusions	All participants were included if they provided metropolitan statistical area locations.
Non-participation	Non-participation is not applicable since no participants were directly recruited to take implicit association tests.
Randomization	Participants were not separated into experimental groups

Reporting for specific materials, systems and methods

We require information from authors about some types of materials, experimental systems and methods used in many studies. Here, indicate whether each material, system or method listed is relevant to your study. If you are not sure if a list item applies to your research, read the appropriate section before selecting a response.

Materials & experimental systems

n/a	Involved in the study
☒	☐ Antibodies
☒	☐ Eukaryotic cell lines
☒	☐ Palaeontology and archaeology
☒	☐ Animals and other organisms
☒	☐ Clinical data
☒	☐ Dual use research of concern
☒	☐ Plants

Methods

n/a	Involved in the study
☒	☐ ChIP-seq
☒	☐ Flow cytometry
☒	☐ MRI-based neuroimaging
